# Differences and diversity of autoimmune encephalitis in 77 cases from a single tertiary care center

**DOI:** 10.1186/s12883-019-1501-5

**Published:** 2019-11-06

**Authors:** Abhinbhen W. Saraya, Kanthita Worachotsueptrakun, Kritchai Vutipongsatorn, Chanikarn Sonpee, Thiravat Hemachudha

**Affiliations:** 1 King Chulalongkorn Memorial Hospital-The Thai Red Cross Society, Thai Red Cross EID-Health Science Center, Bangkok, Thailand; 2 Thai Red Cross EID-Health Science Centre, Bangkok, Thailand; 3 Division of Neurology, Department of Medicine, Faculty of Medicine, Chulalongkorn University, Rama IV Road, Pathumwan, Bangkok, 10330 Thailand

**Keywords:** Encephalitis, Autoimmune encephalitis, Paraneoplastic encephalitis, Limbic encephalitis

## Abstract

**Background:**

The incidence of autoimmune encephalitis has risen globally. There are two general categories of disease-associated antibodies that can be tested for: neuronal surface and intracellular. However, testing both groups of autoantibodies are costly. This study aims to identify differences between groups by comparing clinical presentations, radiological findings and CSF profile of patients, and determine if any parameters are indicative of one group of autoantibodies over another. Additionally, we aim to report the local incidence of less common groups of disease-associated antibodies as well.

**Methods:**

Seventy-seven records of autoimmune encephalitis/encephalomyelitis patients admitted to King Chulalongkorn Memorial Hospital, Bangkok, Thailand, between October 2010 and February 2017 were reviewed. Patients with infections or those with classic central nervous system demyelinating features were excluded.

**Results:**

Of 77 patients, 40% presented with neuronal surface antibodies and 33% had intracellular antibodies. The most common autoantibody detected in each group was anti-NMDAr antibody (25/31, 81%) and anti-Ri antibody (7/25, 28%) respectively. In the neuronal surface antibody group, behavioral change was the most common complaint (45%), followed by seizures (39%) and abnormal movements (29%). In the latter group, seizure was the most common presenting symptom (32%), followed by motor weakness (20%), behavioural change (16%) and abnormal movements (16%). Patients with neuronal surface antibodies were younger (35 vs 48 years old, *p* = 0.04) and more likely to present with behavioral change (45% vs 16%, *p* = 0.02). Mortality rate was higher in the intracellular group (16% vs 3.2%, *p* = 0.09). No differences were detected in magnetic resonance imaging (MRI) and CSF profile.

**Conclusions:**

In the early stages of the disease, both groups have comparable clinical outcomes. Although there were significant differences in age and percentage of patients with behavioral change, both groups of autoimmune encephalitis still shared many clinical features and could not be distinguished based on MRI and CSF profiles. Therefore, we recommend that patients with features of autoimmune encephalitis should be screened for both the neuronal surface and intracellular antibodies regardless of clinical presentation.

## Background

Autoimmunity is a major cause of encephalitis. It is now as common as infectious etiology. Since the discovery of N-methyl-D-aspartate receptor (NMDAr) antibody by Dalmau et al. in 2007 [1], the incidence of autoimmune encephalitis has been rising globally from 0.4/100,000 person-year (1995–2005) to 1.2/100,000 person-year (2006–2015) [2–7]. Additionally, with several novel neuronal antibodies discovered recently such as LGI1, CASPR2, GABA-A/Br, anti-dopamine2 receptor, anti-DPPX, anti-IgLON5 and neurexin-3α [8–15], it is plausible that the diagnosis of autoimmune encephalitis will continue to rise.

Antibodies responsible for autoimmune encephalitis are broadly classified into two categories: neuronal surface and intracellular antibodies [16]. The neuronal surface group comprises of antibodies to surface receptors and protein complexes such as NMDAr, AMPAr, CASPR2, LGI-1 and GABAr [17, 18]. On the other hand, the intracellular group (classic paraneoplastic antibodies), consists of antibodies against intracellular antigens (e.g., Hu, Ri, Yo and CV2) [19].

Several methods can be used to identify these autoantibodies in the serum and cerebrospinal fluid (CSF). In autoimmune encephalitis, techniques such as immunoblotting, immunohistochemistry, and immunocytochemistry with immunofluorescence assays are most commonly used [20]. However, these assays are time-consuming and costly especially if several panels of autoantibodies are tested. Therefore, by comparing the clinical data, radiological results and CSF profiles of patients, we hope to identify key differences that will determine which set of autoantibodies patients are likely to have in order to eliminate unnecessary tests. This would free up time and resources that can be spent on other aspects of healthcare. Additionally, the results could be applicable to other developing countries who could benefit from this cost-saving strategy. Furthermore, this study also aims to report the local incidence and clinical presentations of other less common disease-associated antibodies groups.

## Methods

### Participants

Seventy-seven records of patients with autoimmune encephalitis/encephalomyelitis admitted to KCMH between October 2010 and February 2017 were reviewed.

### Selection criteria

Patients with infectious encephalitis and those with classical demyelinating features were excluded from this study. Patients were included if they met the criteria for encephalitis, which is one or more of the following presentations [21]: cognitive disturbance, behavioral change, focal neurological abnormalities and seizures plus the detection of well-defined antibodies against cell-surface, synaptic, or intracellular proteins in serum or CSF. According to the criteria proposed by Graus et al., antibody negative autoimmune encephalitis was diagnosed in patients who had rapid progression of working memory deficits, altered mental status, or psychiatric symptoms and absence of well-characterized autoantibodies in serum and CSF [22]. The diagnosis of neuro-psychiatric lupus erythematosus (NPLE) was initially determined by clinical assessment, followed by positive results of either ANA, anti-ribosomal P, anti-cardiolipin or lupus anticoagulant in serum [23]. The diagnosis of Hashimoto’s encephalitis comprised of encephalopathy with seizures, myoclonus, hallucinations or stroke-like episodes and subclinical or mild overt thyroid disease with positive serum thyroid antibodies [22]. We used the descriptions of psychopathological features according to Al-Diwani et al. [24] in our study. “Behavior” included agitation, aggression, disorganisation, incoherent speech, violence, incongruent laughter/crying, disinhibition, hyper-religiosity, impulsivity and talking to self. “Catatonia” referred to mutism, stupor, verbigeration and waxy flexibility.

### Investigations

Routine laboratory results were reviewed. These included complete blood count, blood urea nitrogen, creatinine, serum electrolytes, HIV antibody, chest x-ray and liver function tests. Blood cultures were performed in every febrile case. Serum and/or CSF were also collected for autoantibody screening. Autoantibodies panel tests were routinely used in the suspected cases since 2014. The specimens from 2010 to 2013 were also retrospectively tested and reported [2]. Indirect immunofluorescence (IIF) autoimmune panel consisted of anti-NMDAr, anti-AMPAr-1 and 2, anti-CASPR2, anti-LGI-1, anti-GABAr-A and B and DPPX (EUROIMMUN®). The IIF paraneoplastic (PNS) panels and immunoblot anti-neuronal IgG profile comprised of anti-Hu (ANNA-1), anti-Ri (ANNA-2), anti-Yo (PCA-1), PCA-2, anti-Tr, anti-MAG, anti-myelin, anti-GAD, anti-CV2, anti-PNMA2, anti-ampiphysin, anti-neuroendothelium, anti-GFAP, anti-synaptophysin and AGNA/anti-SOX1 (EUROIMMUN®). After collection, the sample was diluted 10-fold and incubated for 30 min at room temperature on slides containing either specific antigen-expressing HEK cells or tissues that naturally expressed these antigens (e.g., cerebellum, pancreas, intestine and nerve cell). They were then incubated in secondary antibody conjugated with fit-C fluorescence and observed under an inverted fluorescence microscope. As for the immunoblot assay (EUROLINE®), specimen was diluted accordingly (1:101 for anti-neuronal) put on the test strip and incubated for 30–120 min. After a wash, the strip was incubated with conjugated enzyme and substrate before being evaluated by EUROLineScan®. The IIF assay for aquaporin-4 (AQP4) antibody, anti-thyroid peroxidase and anti-thyroglobulin was also conducted in some cases based on clinical suspicion.

All patients were screened for tumor by computed tomography (CT) of chest and whole abdomen including pelvis. Pelvic examination and ultrasonography were performed in female patients with anti-NMDAr and anti-Yo. Mammogram was also indicated in some cases based on clinical judgement. Modified Rankin Scale (mRS) was used to evaluate patients before discharge.

### Statistics

SPSS software version 17 was used to describe and compare epidemiological data between neuronal surface and intracellular groups. Categorical data were analyzed using chi-square and Fisher’s exact test. Mann-Whitney U test was performed to analyze numerical data. *P*-value of less than 0.05 was considered statistically significant.

## Results

**Fig. 1:**
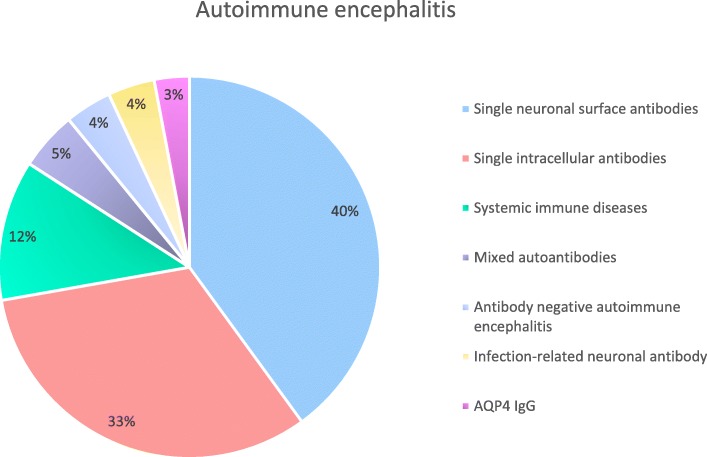
Proportion of each groups of autoimmune encephalitis

Seventy-seven patients in this study were from 24 provinces nationwide with 50 cases from the central region of Thailand. Thirty-one cases (40.3%) presented with neuronal surface antibodies while 25 cases (32.5%) had intracellular antibodies. 28% of patients belonged to the systemic autoimmune diseases (NPLE and Hashimoto’s encephalopathy), coexisting neuronal antibodies, antibody-negative autoimmune encephalitis, infection-related neuronal antibody positive group (syphilis and *Listeria monocytogenes* infections) and atypical AQP4-IgG, which presented with encephalitis syndrome (Fig. 1). The prevalence and characteristics of autoantibodies from each group of autoimmune encephalitis are described in Fig. 1 and Table 1.

**Table 1:** Description of autoantibodies seen in neuronal surface antibody group, intracellular antibody group and coexisting-antibodies group

Antibodies	Number of serum positive patients	Number of CSF positive patients	Age range (year)	% Male	Presenting symptoms	Evidence of tumor at 1st screening (cases)
Anti-NMDAr (25 cases)	24	16 (8 N/A)	5–79	44.1	Behavior, psychosis, dyskinesia, seizures, memory impairment, catatonia, mood disorders	Ovarian tumor [3]
Anti-AMPAr (2 cases)	2	1 (1 N/A)	2–72	50.0	Behavior, stiff-person syndrome	NSCLC [1]
Anti-GABA-Br (2 cases)	2	1 (1 N/A)	42–70	50.0	Behavior, seizures, myoclonus	Stomach cancer [1]
Anti-LGI1 (2 cases)	2	1 (1 N/A)	67–75	50.0	Behavior, seizures, FBDS	None
Anti-Ri (ANNA2) (7 cases)	7	1 (6 N/A)	44–82	42.9	Seizures, psychosis, dyskinesia, weakness	None
Anti-Yo (PCA1) (3 cases)	3	1 (2 N/A)	11–31	66.7	Seizures, diplopia, drowsiness, weakness	Germ cell tumor [1], high CA-125 (1)
Anti-PNMA2 (3 cases)	3	1 (1 N/A)	35–80	66.7	Behavior, dyskinesia, rapidly progressive dementia, weakness	Pancreatic cancer [1],high CA-125 (1)
Anti-recoverin (3 cases)	3	0	40–71	66.7	Behavior, ataxia, numbness	None
Anti-CV2 (CRMP5) (2 cases)	2	1 (1 N/A)	59–65	50.0	Psychosis, memory impairment	Ovarian cancer [1]
Anti-Hu antibody (2 cases)	2	N/A	28–29	0.0	Behavior, seizures, weakness, numbness	None
Anti-GAD65 (2 cases)	2	1 (1 N/A)	1–16	50.0	Seizures, opsoclonus-myoclonus, ataxia	Neuroblastoma [1]
Anti-SOX1 (2 cases)	2	2	33–66	50.0	Behavior, dyskinesia	None
Anti-titin (1 case)	1	0	86	0:0	Drowsiness, psychomotor retardation	None
Anti-NMDAr +ANA (2 cases)	2	2	15–30	0:0	Behavior, psychosis, seizures	Ovarian tumor [1]
Anti-NMDAr +anti-Ri (1 case)	1	N/A	18	100.0	Seizures	None
Anti-NMDAr +AQP4 (1 case)	1	1	24	0.0	Behavior, dyskinesia, weakness	None

*NSCLC* non-small cell lung cancer, *FBDS* fasciobrachial dystonic seizure, *N/A* data not available

### Neuronal surface antibody group

Anti-NMDAr antibody is the most common antibody in the neuronal surface group (25/31 cases, 81%). Most patients (28 cases, 90.3%) had encephalitis with two and one cases of myelitis and encephalomyelitis respectively. The median age and range are shown in Table 2. Half of this group (16 cases, 51.6%) had no underlying diseases. Four patients were previously diagnosed with anti-NMDAr encephalitis with median recurrent time of 287.5 days (ranged from 61 to 456 days). One patient had HIV infections with CD4 count less than 200 cells/mm.^3^ A quarter of the patients (8 cases, 25.8%) also presented with prodromal symptoms such as headache and/or fever. However, they were absent in the remaining patients (23 cases, 74.2%).

**Table 2:** Comparison between neuronal surface antibody group and intracellular antibody group

Characteristics	Neuronal surface antibodies (*n* = 31)	Intracellular antibodies (*n* = 25)	*P* value
Baseline			
Gender			0.83
- male	14 (45.2%)	12 (48%)	
- female	17 (54.8%)	13 (52%)	
median age (years)	30.5 (2–79)	45.5 (1–86)	**0.04**
Presenting symptoms			
Behavior	14 (45.2%)	4 (16%)	**0.02**
Psychosis/mood	7 (22.6%)	3 (12%)	0.30
Seizures	12 (38.7%)	8 (32%)	0.60
Abnormal movements (total)	12 (38.7%)	6 (24%)	0.24
Generalized dyskinesia/chorea	5 (16.1%)	3 (12%)	0.66
CSF profile			
median CSF white blood cell (cells/mm^3^)	2 (0–82)	1 (0–31)	0.12
median CSF protein (mg/dl)	26.5 (11–242)	44 (2–440)	0.27
median CSF glucose (mg%)	65.5 (44–149)	67 (42–95)	0.46
Duration of disease onset to treatment (days)	15 (1–420)	20 (1–700)	0.385
Median hospital stay (days)	23 (4–150)	31.5 (5–126)	0.57
Outcome at discharge from hospital			
Completely recovered (mRS 0–1)	3 (9.7%)	0 (0%)	0.25
Partially recovered (mRS 2–3)	20 (64.5%)	15 (60%)	0.73
Disable (mRS 4–5)	7 (22.6%)	6 (24%)	0.9
Dead	1 (3.2%)	4 (16%)	0.16

*mRS* modified Rankin Scale, *P* value <0.05 set in bold is considered statistical significant

Behavioral change was the most common presenting complaint with 14 cases (45.2%), followed by seizures (12 cases, 38.7%) and abnormal movements (9 cases, 29.0%) (Table 2). Among the nine patients with abnormal movements, five had chorea/dyskinesia (all had anti-NMDAr with one patient also presented with catatonia), two had faciobrachial dystonic seizures (both had anti-LGI1), one had stiff-person syndrome (anti-AMPAr-2), and one had myoclonus (anti-GABAr).

CSF examination was obtained from 27 patients in this group. There were ten cases of CSF pleocytosis (37.0%) and all had CSF white cell count below 100 cells/mm^3^. Most of the remaining cases had normal CSF protein and glucose level. Only one patient (anti-NMDAr) had a high CSF protein level of 242 mg/dl. Neuroimaging data were available in 27 cases. The majority presented with normal and non-specific findings (17 cases, 63.0%). The most common abnormality in brain MRI was increased signal intensity in T2-weighted image at the temporal lobe (3 cases, 11.1%). Other findings were leptomeningeal enhancement, subcortical, basal ganglia and multifocal lesions. In the neuronal surface antibody group, three cases of tumor (9.7%) were found on admission or during the first follow-up (Table 1).

Fourteen patients (45.2%) received immunotherapy (IVIg 2 g/kg/course or plasmapheresis 5 cycles) and 18 patients (58.1%) were given intravenous methyl prednisolone 1 g/kg/day for 5 days. Tumors were appropriately treated upon discovery. Median length of stay in the hospital was 24 days (ranged from 4 to 150 days). Seven patients (22.6%) had poor outcome at discharge from hospital (mRS score 4–5) and one died from hospital acquired infection.

### Intracellular antibody group

Anti-Ri was the most common autoantibody detected in the intracellular antibody group (7/25 cases, 28%). Eleven (44.0%) had no underlying disease, four had SLE, one had chronic HIV infections and one was previously diagnosed with malignant tumor (germ cell tumor).

As seen in Table 2, seizure was the most common presenting symptom (8 cases, 32.0%), followed by motor weakness (5 cases, 20.0%) and. Behavioral change (4 cases, 16.0%). There were four cases (16.0%) of abnormal movements: one opsoclonus-myoclonus (anti-GAD65) and three generalized chorea (anti-Ri and anti-Sox1). Memory impairment was the first presenting complaint in two patients (8.0%). Six patients (24.0%) had tumors or evidence of malignancy with two patients presented with high blood level of CA-125 (anti-Yo, PNMA2) and the other four patients each presented with germ cell tumor (anti-Yo), ovarian tumor (anti-CV2), neuroblastoma (anti-GAD) and pancreatic cancer (PNMA2).

Neuroimaging data were available in 22 patients. Nine (40.9%) had normal or non-specific lesions. Predominantly increased signal intensity lesions in a T2-weighted image and FLAIR image at medial temporal lobe was found in three patients (13.6%). Other lesions were found in the cerebral cortex, subcortical white matter, basal ganglia, thalamus, brainstem and cerebellum. CSF examination was performed on 21 patients. Five (23.8%) had a white blood cell count of more than 5 cells/mm^3^, fifteen (71.4%) had a protein level of less than 100 mg/dl and all had a normal glucose level.

Eight patients (32.0%) received IVIg 2 g/kg/course and seven (28.0%) were given IV methyl prednisolone 1 mg/kg/day for 5 days. Median length of stay in the hospital was 31 days (range 5–126). Six patients (24.0%) had poor outcome at discharge from hospital (mRS score 4–5) and four (16.0%) died. The causes of death were hospital acquired pneumonia (2 cases) and septicemia (2 cases).

### Systemic autoimmune encephalitis group

There were nine cases in the systemic autoimmune encephalitis group with six resulted from neuropsychiatric lupus erythematosus (NPLE). The median age of patients in this group was 33 (range 17–67) and most of the cases were female (88.9%). Four patients had been diagnosed with SLE prior to admission and two had underlying thyroid disease. Two cases presented with myelitis, while the remaining seven had encephalitis symptoms such as behavioral changes, seizure, psychosis, memory impairment and altered consciousness. Most patients in this group had normocellular CSF with normal CSF glucose level. Only three cases had a CSF protein level of over 60 mg/dl. Six patients underwent brain MRI and half of them presented with normal or non-specific findings. Others showed increased signal intensity lesions in a T2-weighted image and FLAIR image at the cortical area, subcortical white matter and midline structures. No evidence of tumor was detected in this group. A large proportion of patients (44.4%) received high dose corticosteroids for treatment with a median hospital stay of 20 days. In terms of outcome at discharge from hospital, four patients partially recovered while the other four became disabled. One patient with NPLE had died.

### Mixed autoantibodies group

There were four cases of mixed autoantibodies with all four presented with anti-NMDAr antibody and one additional autoantibody (Table 1). Neuropsychiatric lupus was diagnosed in two patients who both had systemic SLE and ANA level of 1:2560. They also presented with behavioral change and psychosis. Neuroimaging was unremarkable except for the patient with coexisting NMO antibody who had lesions at the midline and cortical structures.

## Discussion

### Comparison between local population and other studies

This study described the clinical, radiological and laboratory findings in patients with autoimmune encephalitis diagnosed and treated at a tertiary care hospital in Bangkok, Thailand. Antibodies against neuronal surface antigen were the most common followed by those against intracellular antigen (46 and 38%, respectively). The most common autoimmune encephalitis in this study was anti-NMDAr encephalitis. This is consistent with other studies, which report that anti-NMDAr encephalitis is the most prevalent immune-mediated encephalitis worldwide to date [25, 26]. Some reports found that anti-LGI1 was presumed to be the second most common cause of autoimmune encephalitis among the neuronal surface antibodies group [12, 27]. However, we only identified two cases of anti-LGI1 encephalitis among 31 cases (6%). This is likely because LGI1 antibodies were more common among elderly patients with antibody-associated central nervous system (CNS) syndrome [28, 29], while the majority of patients in our study were middle-aged (mean age of 40). Both cases of anti-LGI1 encephalitis in our study had FBDS that responded to immunotherapy rather than anti-epileptic drugs, which is similarly reported by Thompson et al. [30]. Additionally, these two patients were more than 50 years old and only had CNS manifestations—a finding that is consistent with Gadoth et al. [31].

Among the two cases of anti-AMPAr antibody encephalitis, one had SCLC, which was the most commonly associated tumor [32]. The other patient, a 2-year-old girl with no malignancy detected, presented with a stiff-person syndrome. She eventually recovered after receiving baclofen and supportive treatment. Her serum anti-AMPAr antibody was subsequently negative at a 3-month-follow up.

There are some differences in this study and previously published literature. For instance, in the neuronal surface group, only 26% of our cohort had presented with prodromal symptoms while some studies have suggested that this figure could be as high as 61–70% [25, 33, 34]. Additionally, CSF pleocytosis was detected in 37% of our patients compared to 80% in a previous review [33].

There was a strong association between anti-NMDAr encephalitis and ovarian tumor/teratoma [1, 35, 36], especially among Asian and African-American women where 50% of adult female patients were estimated to have ovarian teratoma [37]. However, in our study, only two cases (9%) of ovarian tumor were found at the first immediate tumor screening despite a thorough investigation with abdominal and pelvic CT and pelvic ultrasonography. Interestingly, this is consistent with anti-NMDAr encephalitis case series in China [25] and Korea [38], which found that ovarian tumor was present in four (8%) and two (9%) patients respectively. However, since paraneoplastic neurological syndromes can present within 5 years of the initial diagnosis [39], annual tumor screening should be performed in the remaining cases. Furthermore, oro-facial dyskinesia was the most common movement disorder among anti-NMDAr encephalitis patients in our study, which is consistent with previous studies [26, 35, 40]. However, we did not find dystonia as common as suggested by Varley et al. [41] and there was only one case of catatonia in our study.

There were three patients (4%) that fit the criteria of autoantibody negative autoimmune encephalitis. This raises a question of whether there is truly no antibody or if there is an unknown neuronal antibody that we could not detect with the current tests used in our study.

Finally, we discovered that some patients had non-classical paraneoplastic autoantibodies such as anti-recoverin, anti-titin and anti-SOX1, which were usually not considered as the cause of paraneoplastic CNS disease. Anti-recoverin is one of the causes of cancer-associated retinopathy [42]. Here, we found three cases of anti-recoverin associated encephalitis that presented with behavioral symptoms. Anti-titin and anti-SOX1 often present with other classical paraneoplastic antibodies (anti-Hu, Ri, Yo etc.) in paraneoplastic syndrome of the nervous system [43, 44]. Since no other coexisting autoantibodies were identified in our cases, these antibodies may be directly responsible for the neurological symptoms seen.

### Comparison between neuronal surface and intracellular groups

Since the majority of autoimmune encephalitis patients in our study belonged to either the neuronal surface group or the intracellular group, the differences between these two groups were summarized in Table 2.

Patients in the intracellular group were significantly older than those in the neuronal surface group (48 vs. 35 years old, *p* = 0.04). Furthermore, behavioral change, which was the third most common symptom in the intracellular group, was significantly more common in the neuronal surface group as well (45% vs. 16%, *p* = 0.02). On the other hand, seizure was the most common complaint in the intracellular group, but there were no significant differences in its prevalence between the two groups (32% vs. 39%, *p* = 0.60). Abnormal movements and psychiatric features were more common in neuronal surface group, but similarly there were no significant differences (*p* = 0.24 and 0.30 respectively). Additionally, the intracellular group tended to have a longer duration of symptoms before admission to hospital (3 months vs 2 months).

As for CSF profile, the intracellular group had a large proportion of normocellular CSF (76%). Neuroimaging (MRI) data could not distinguish between these two groups of autoantibody-mediated encephalitis.

The recovery rate and morbidity at discharge from hospital between both groups were comparable. The mortality rate was noticeably higher in the intracellular group although this was not statistically significant (16% vs. 3.2%, *p* = 0.16). Therefore, it seems that at least in the early stages of the disease, patients with surface and intracellular antibodies have similar outcome. However, further studies with more participants and higher power are needed to confirm this result.

## Conclusion

We described different groups of autoimmune encephalitis patients in Thailand. The two most prevalent groups were neuronal surface and intracellular groups, with the former being more common than the latter. However, in the earlier stages of the disease, both groups have comparable clinical outcomes. Even though the age of onset and percentage of patients with behavioral change were statistically different between these two groups, they still shared many clinical features and could not be distinguished based on radiological findings and CSF profiles. As such, we recommend that patients with suspected autoimmune encephalitis should be screened for both the neuronal surface and intracellular antibodies.

## Data Availability

Data used and/or analyzed in this study are available from the corresponding author on reasonable request.

## Abbreviations

AGNA: antiglial nuclear antibodies
AMPAr: alpha-amino-3-hydroxy-5-methyl-4-isoxazolepropionic acid receptor
ANNA: anti-neuronal nuclear antibody
AQP-4: Aquaporin-4
CASPR2: contactin-associated protein-like 2
DPPX: dipeptidyl-peptidase–like protein 6
FBDS: faciobracial dystonic seizure
FLIAR: Fluid-attenuated inversion recovery
GABAr: gamma-aminobutyric acid receptor
GAD: glutamic acid decarboxylase
GFAP: Glial Fibrillary Acidic Protein
LGI-1: Leucine-rich glioma-inactivated 1
MAG: myelin-associated glycoprotein
NMDAr: N-methyl-D-aspartate receptor
PCA: Purkinje cell antibody
SCLC: small cell lung cancer
